# Awareness and Feasibility of Women Chairing Cardiology Sessions in Scientific Meetings: A Nationwide Survey by the Japanese Circulation Society

**DOI:** 10.3389/fcvm.2022.871546

**Published:** 2022-06-03

**Authors:** Atsuko Nakayama, Chizuko A. Kamiya, Sachiko Kanki, Tomomi Ide, Yasuko K. Bando, Yukari Uemura, Yayoi Tetsuou Tsukada

**Affiliations:** ^1^Department of Cardiovascular Medicine, The University of Tokyo, Tokyo, Japan; ^2^Department of Cardiovascular Medicine, Sakakibara Heart Institute, Fuchu, Japan; ^3^Department of Obstetrics and Gynecology, National Cerebral and Cardiovascular Center, Osaka, Japan; ^4^Department of Thoracic and Cardiovascular Surgery, Osaka Medical and Pharmaceutical University, Osaka, Japan; ^5^Department of Cardiovascular Medicine, Graduate School of Medical Sciences Kyushu University, Fukuoka, Japan; ^6^Department of Cardiology, Nagoya University Graduate School of Medicine, Nagoya, Japan; ^7^Center for Clinical Sciences, National Center for Global Health and Medicine, Tokyo, Japan; ^8^Department of General Medicine and Health Science, Nippon Medical School, Tokyo, Japan

**Keywords:** women, sex, gender, diversity, cardiologist, session chair

## Abstract

**Background:**

Diversity and inclusion remain a concern in the field of cardiology. Female cardiologists have less opportunity to chair sessions in scientific meetings than men. However, cardiologists’ awareness and perspectives on feasibility of chairing sessions is poorly understood.

**Methods and Results:**

A web-based survey on awareness regarding the commitment of chairing sessions was sent to 14,798 certificated cardiologists registered with the Japanese Circulation Society (JCS). A total of 3,412 valid responses were obtained, such as 523 women and 2,889 men. Female cardiologists exhibited less interest in serving as chairpersons in Japanese and English sessions (71% women vs. 82% men, *p* < 0.001, 30% women vs. 40% men, *p* < 0.001). Influencing factors of chair acceptance in Japanese sessions for female cardiologists were being a cardiologist for over 10 years [odds ratio (*OR*) 1.84, 95% confidence interval (*CI*) 1.02–3.33], experience studying abroad (OR 3.35, 95% *CI* 1.93–5.81) and chairing sessions (*OR* 8.39, 95% *CI* 5.48–12.9), having a Doctor of Philosophy (*OR* 2.82, 95% *CI* 1.09–7.31), presence of 4 or more female cardiovascular specialists in the hospital (*OR* 1.70, 95% *CI* 1.10–2.61) and of role models (*OR* 2.86, 95% *CI* 1.93–4.24), and awareness of the JCS chairperson’s manual (*OR* 10.7, 95% *CI* 6.67–17.1). The receiver operating characteristic (ROC) curve revealed that the number of female cardiovascular specialists in a hospital was a more sensitive predictor of chair acceptance among male than female cardiologists.

**Conclusions:**

Female cardiologists were less likely to accept chairing sessions compared with male cardiologists and the presence of female cardiovascular specialists positively influenced chair acceptance.

## Introduction

As society matures, the symbiosis of diversity becomes increasingly important. Diversity and inclusion (D/I) focuses not only on gender but also race, age, and other aspects of life to improve the world. Due to historical and cultural backgrounds, the acceptance of diversity in Japan has been hindered compared to other developed countries (1).

The Japanese Circulation Society (JCS) had 26,645 members as of June 2020 and annual host’s large medical academic conferences in Japan, such as 18,600 participants in 2020 (2). However, there have been few female chairpersons, large restricted to senior physicians, and researchers. Since 2011, the JCS has implemented D/I action and independently used the D/I session chair ratio as an indicator of leadership allocation (3).

In addition, the JCS–Josei Junkanki Consortium (JCS–JJC) released a chairperson’s manual (4). As the format of the academic conference recently changed due to the corona virus disease-19 (COVID-19) pandemic and information technology (IT) development, shifting to an online format, the feasibility of chairing sessions may be changing (5, 6). Although studies have examined female presenters in academic societies (7), research on feasibility of chairing sessions in academic meetings is lacking.

Keeping this in mind, this study aimed to examine the factors influencing session chair acceptance for Japanese and English sessions among cardiologists across gender.

## Materials and Methods

### Study Design

A qualitative survey was conducted by our research team of the Committee for Diversity Promotion of the JCS to investigate cardiologists’ perspectives on chairing sessions. The questionnaire was emailed to 14,798 medical doctors who were the JCS members also. Doctors belonged to the 1,408 non-duplicate hospitals which covered 92% of all active cardiovascular hospital in Japan (8) responded to this survey. Data were obtained from 3,412 doctors (23% response rate), with all personal information removed. Because we used the Google form for the questionnaire, all responses were valid responses which replied for all questions in this survey.

The process of how JCS decides who serves as chair in the JCS meetings was according to the recommendation by the councilors of JCS from a pool of expert lists. Subsequently, session chairs were selected after confirming the acceptance of the candidates who received the offering email from JCS office. For the annual meeting of JCS (2021), the Committee for Diversity Promotion of the JCS made the list of chair candidates who have cardiovascular specialist qualifications and accept a chair, for the councilors with recommendation of the positive invitation to female doctors to a chair.

This study was conducted in accordance with the ethical principles of the Declaration of Helsinki. Further, the study design was approved by the JCS Ethics Committee (ID: 14). Informed consent was obtained from all patients according to the protocol approved by the JCS Ethics Committee.

### Doctor’s Degree

In Japan, students usually become physicians after graduating from high school and medical school continuously and then passing the national examinations of Doctor of Medicine (M.D.). Doctor of Philosophy (Ph.D.) is degree for a physician after completing a doctoral course in a medical graduate. Ph.D. is the higher degree of M.D. for a physician in Japan.

### Outcomes

The main aim of investigating the acceptance rate of Japanese or English session chairs was to determine whether significant differences in chair acceptance were seen between male vs. female cardiologists.

### Statistical Analysis

The cardiologist’s characteristics were compared using the χ2-test for non-continuous variables, unpaired *t*-test for normative continuous data, using SPSS v22 (IBM Inc., Armonk, NY, United States). Logistic regression analysis was used to analyze the contribution of each factor to the chair’s acceptance, while the analysis was carried out using interactive P was used to measure whether the strength of each factor within male vs. female group was heterogeneous. We performed a receiver operating characteristic (ROC) curve analysis to explore whether the number of female cardiovascular specialists belonging to each hospital could be a prospective marker for chair acceptance. The significance level was set at the alpha level of 0.05.

## Results

### Background of Respondents

**FIGURE 1:**
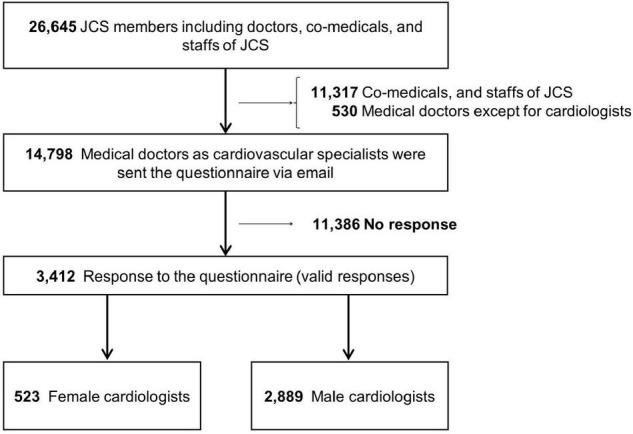
Flowchart of response to the questionnaire. The JCS had 26,645 members such as doctors, co-medicals, and staffs of JCS. The 14,798 doctors in the cardiovascular field were registered until April 2021 in JCS (2021) and were sent the questionnaire *via* email at April 28, 2021. Data were obtained from 3,412 doctors. Of the 3,412 responses, 523 were from women and 2,889 from men.

Of the 3,412 valid responses (Figure 1), 523 were from women and 2,889 from men (Table 1). A total of 3,406 respondents (99.8%) had cardiovascular specialist qualifications.

**TABLE 1:** Respondent characteristics.

	Female cardiologists	Male cardiologists	*p*-value
Total number, *n* (%)	523	2889	
Age, years ± SD	47 ± 8	50 ± 9	< 0.001
Years as a doctor, years ± SD	21 ± 8	25 ± 9	< 0.001
Years as a cardiologist, years ± SD	18 ± 9	22 ± 11	< 0.01
Qualified Ph.D., *n* (%)	386 (74%)	2292 (79%)	0.14
JCS fellow, *n* (%)	25 (5%)	259 (9%)	< 0.01
Study abroad experience, *n* (%)	127 (24%)	1125 (39%)	< 0.001
Experience as session chairperson, *n* (%)	359 (69%)	2470 (86%)	< 0.001
Chair acceptance in Japanese sessions, *n* (%)	371 (71%)	2378 (82%)	< 0.001
Chair acceptance in English sessions, *n* (%)	155 (30%)	1142 (40%)	< 0.001
JCS session chairperson acquaintances, *n* (%)	446 (85%)	2419 (84%)	0.21
Presence of role models, *n* (%)	348 (67%)	2041 (71%)	0.03
Only female role models, *n* (%)	119 (23%)	11 (0%)	< 0.001
Only malerole models, *n* (%)	60 (12%)	1714 (59%)	
Bothrole models, *n* (%)	163 (31%)	324 (11%)	
Childcare duties, *n* (%)	297 (57%)	1426 (49%)	0.001
Preschool child, *n* (%)	76 (15%)	289 (10%)	< 0.001
Juvenile child, n (%)	59 (11%)	449 (16%)	
Preschool and juvenile children, *n* (%)	36 (7%)	238 (8%)	
Childcare support by the conference			
Need childcare support during the session, *n* (%)	210 (40%)	1329 (46%)	< 0.001
Do not need childcare support during the session, *n* (%)	103 (20%)	330 (11%)	
Need not to ask for childcare support during the session, *n* (%)	93 (18%)	671 (23%)	
Social childcare service			
Social childcare service allows for chairperson duties, *n* (%)	76 (15%)	233 (8%)	< 0.001
Social childcare service does not allow for chairperson duties, *n* (%)	52 (10%)	174 (6%)	
Need not to ask for social childcare service, *n* (%)	240 (46%)	1784 (62%)	
Awareness of the JCS–JJC chairperson’s manual, *n* (%)	281 (54%)	1140 (40%)	< 0.001
The JCS–JJC chairperson’s manual was useful*, *n* (%)	257 (91%)	1034 (91%)	< 0.001
The JCS–JJC chairperson’s manual was helpful for chair acceptance, *n* (%)	429 (82%)	2168 (75%)	< 0.01

*Rate was calculated among manual readers. JCS, Japanese circulation society; JJC, Josei Junkanki Consortium; Ph.D., Doctor of Philosophy.

**FIGURE 2:**
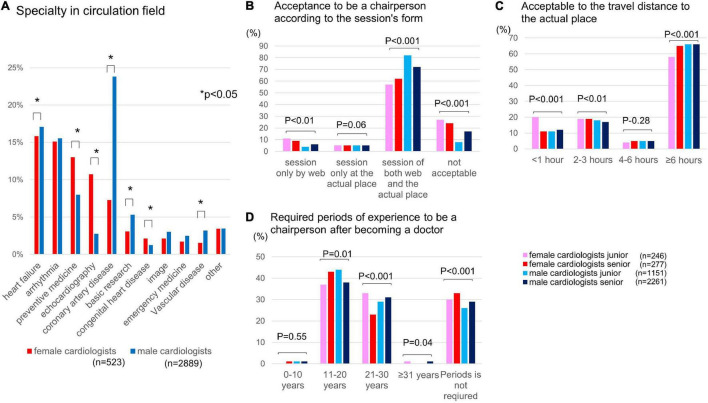
Specialty of cardiologists in Japan, awareness of chair acceptance for scientific meetings, and changes in the rates of female cardiologists. **(A)** Background of cardiologist specialties in the cardiovascular field. **(B)** Chair acceptance for scientific meetings. **(C)** Chair acceptance based on travel distance to the meeting. **(D)** Experience required to be a chairperson (years). *p < 0.01.

Significant differences were observed between men and women in the cardiovascular specialty in all fields, except imaging and emergency medicine (Figure 2A). In the fields of preventive medicine, echocardiography, and congenital heart disease, the rate of female cardiologists was higher. However, the rate of female vs. male cardiologists was markedly lower in the field of coronary artery disease (7 vs. 24%, *p* < 0.0001).

**FIGURE 3:**
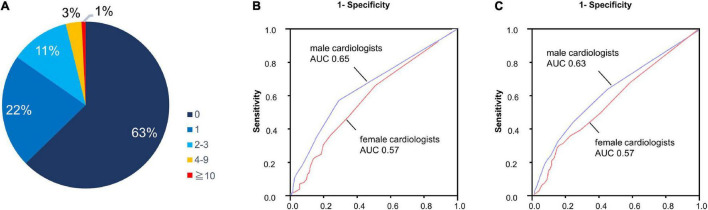
Number of female cardiovascular specialists belonging to each hospital. **(A)** Number of female cardiovascular specialists belonging to each hospital in all registered hospitals (*n* = 1408). **(B)** Acceptance of chairperson by the number of female cardiovascular specialists belonging to each hospital: Japanese session. **(C)** Acceptance of chairperson by the number of female cardiovascular specialists belonging to each hospital: English session.

Of the 1,408 non-duplicate hospitals in this survey, 1,193 (85%) had one or less female specialist, 161 (11%) had two to three, and only 56 (4%) had four or more (Figure 3A). Limited to the 205 academically inclined hospitals, 102 (50%) had one or less female specialist, 58 (28%) had two to three, and 45 (22%) had four or more.

### Preference for Form of Scientific Meetings

A 2 × 2-group comparison by age (younger, <45 years; older, ≥45 years) and gender indicated that younger female cardiologists preferred to participate in online sessions and have a travel time to the meeting of less than 1 h (Figures 2B,C). Most respondents in all groups stated that the experience required to chair sessions was 11–20 years; however, older female cardiologists considered it inappropriate to select a chairperson based on experience, with the lowest proportion answering 21–30 years of experience in the four groups (*p* < 0.001; indicated with a red bar in Figure 2D).

### Factors Influencing Chairperson Acceptance

Female cardiologists had a lower rate of experience studying abroad and chairing sessions compared with male cardiologists (24 vs. 39%, *p* < 0.001, 69 vs. 86%, *p* < 0.001, respectively) and a lower rate of acceptance for Japanese and English sessions (71 vs. 82%, *p* < 0,001, 30 vs. 40%, *P* < 0.001, respectively; Table 1). Female cardiologists had fewer role models than male cardiologists (67 vs. 71%, *p* = 0.03), more childcare duties (57 vs. 49%, *p* = 0.001), and higher awareness of the JCS–JJC chairperson’s manual (54 vs. 40%, *p* < 0.001). In addition, male cardiologists with male role models accounted for 70% (male 59% and both 11%), while 54% (female 23% and both 31%) of female cardiologists had female role models.

### Subgroup Analysis of Chair Acceptance

**FIGURE 4:**
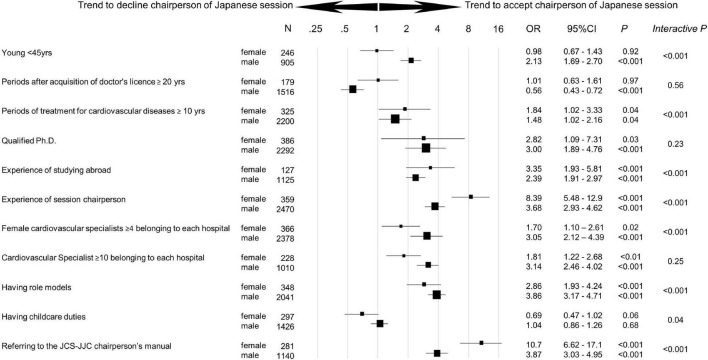
Trend to accept chairperson of Japanese session by gender. Odds ratio [95% confidence interval (*CI*)] for the trend to accept the chairperson of the Japanese session by gender was analyzed in each subgroup. Interactive *p* was used as an index to measure the strength of each factor within male vs. female group. Ph.D., Doctor of Philosophy.

A logistic regression analysis on answers regarding acceptance for chairing Japanese sessions revealed that male cardiologists who were younger [odds ratio (OR) 2.13, 95% confidence interval (CI) 1.69–2.70, *p* < 0.001], had 4 or more female cardiovascular specialists in the hospital (*OR* 3.05, 95% *CI* 2.12–4.39, *p* < 0.0001), and had role models (*OR* 3.86, 95% *CI* 3.17–4.71, *p* < 0.001) were significantly more often accepted as chair (Figure 4). Female cardiologists with 10 or more years of experience in cardiovascular treatment (*OR* 1.84, 95% *CI* 1.02–3.33, *p* < 0.001), who studied abroad (*OR* 3.35, 95% *CI* 1.93–5.81, *p* < 0.0001), who chaired sessions in the past (*OR* 8.39, 95% *CI* 5.48–12.9, *p* < 0.0001), and were aware of the JCS–JJC chairperson’s manual (*OR* 10.7, 95% *CI* 6.62–17.1, *p* < 0.0001) had a higher rate of chair acceptance. Among respondents with childcare needs, female cardiologists tended to decline Japanese session chairs (*OR* 0.69, 95% *CI* 0.47–1.02, *p* = 0.06), whereas male cardiologists didn’t decline (*OR* 1.04, 95% *CI* 0.86–1.26, *p* = 0.68).

**FIGURE 5:**
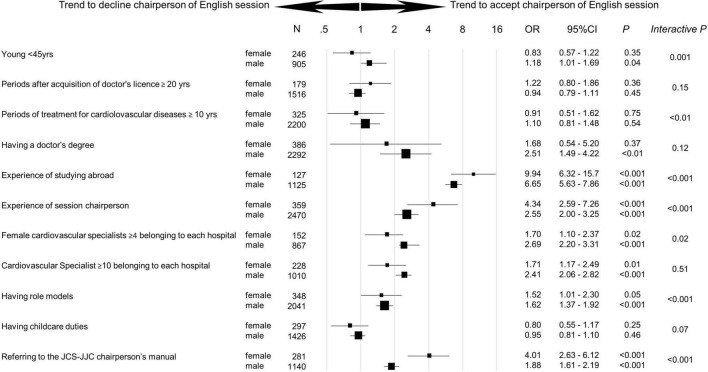
Trend to accept chairperson of English session by gender. Odds ratio [95% confidence interval (*CI)*] for the trend to accept the chairperson of the English session by gender was analyzed in each subgroup. Interactive *P* was used as an index to measure the strength of each factor within male vs. female group. Ph.D., Doctor of Philosophy.

The official language of the session was an important factor that positively affected the decision to accept the role of chairperson. For the English sessions, male cardiologists who were younger (*OR* 1.18, 95% *CI* 1.01–1.69, *p* = 0.04), had 4 or more female cardiovascular specialists in the hospital (*OR* 2.69, 95% *CI* 2.20–3.31, *p* < 0.001), and had role models (*OR* 1.62, 95% *CI* 1.37–1.92, *p* < 0.001) accepted chair (Figure 5). Female cardiologists who studied abroad (*OR* 9.94, 95% *CI* 6.32–15.7, *p* < 0.001), chaired sessions in the past (*OR* 4.34, 95% *CI* 2.59–7.26, *p* < 0.001), and were aware of the JCS–JJC chairperson’s manual (*OR* 4.01, 95% *CI* 2.63–6.12, *p* < 0.001) accepted chairing English sessions more often. In the subgroup with childcare duties, there were no significant differences between male and female cardiologists in the English session chair acceptance rate.

The ROC curve analysis demonstrated that the number of female cardiovascular specialists in the hospital was a more sensitive predictor of chair acceptance among men than women for Japanese and English sessions [area under the curve (AUC) 0.65 vs. 0.57, AUC 0.63 vs. 0.57, respectively] (Figures 3B,C). Limited to the academically inclined hospitals, the number of female cardiovascular specialists was a still more sensitive predictor of chair acceptance among men than women for Japanese and English sessions (AUC 0.65 vs. 0.58, AUC 0.62 vs. 0.59, respectively).

## Discussion

A nationwide survey of cardiologists revealed that women had a lower chair acceptance than men. Acceptance for official language session was significantly higher among men than women for younger cardiologists (<45 years), hospitals with four or more female cardiovascular specialists, and those who had role models. Chair acceptance for English session was significantly higher among women than men for cardiologists with over 10 years of cardiovascular practice, who studied abroad, who chaired sessions in the past, and who were aware of the JCS–JJC chairperson’s manual.

### New Format of Scientific Meetings and Family Duties

During the COVID-19 pandemic, conventional on-site meetings were switched to an online format (5, 6), enabling participation without visiting the conference venue. Nursing and child care duty may disable young cardiologists from participating in onsite conference. The results of the present study indicated that young female cardiologists preferred an online format and were less likely to attend local conferences, perhaps due to parenting duties. Therefore, web-based venues should continue to be partially implemented in the future, even after the pandemic.

The working condition of the female is greatly changed by the existence of marriage, pregnancy, delivery, childcare, and caring for family members with illness or disability in Japan, where the traditional thinking of gender role beliefs strongly remains. Although housework hours are longer for women than men in all countries, focusing on the male-to-female ratio (the ratio of women with men set as 1), Japan has the highest ratio (5.5-fold), followed by 4.4-fold in Korea, and 2.3-fold in Italy among the OECD countries (9).

This survey showed the trend of declining chairing a Japanese session among female than male cardiologists in the subgroup of having childcare duties. Childcare services rapidly developed in Japan (10) and the JCS provides the nursery service for the participants’ pre-school children after the birth of 3 months at the scientific meetings. In Japan, high quality childcare services are provided by both public and private, and many female doctors also use the services. It is expected that even in the academic conference, a temporary childcare center is set up for the participants. In such a background, temporary childcare services would satisfy the participants who need the service; therefore, childcare might not a strong factor for rejecting chairing sessions in this survey.

### Effects of Diversity Promotion

The number of female cardiology specialists in the hospital impacted chair acceptance, with a stronger positive influence on men than women. The acceptance rate to the chair in the hospital where female cardiovascular specialists ≥4 were belonging might be elevated by academically inclined hospitals. However, limited to the academically inclined university hospitals, the more cardiologists accepted chairing a session in the hospitals which more female specialists belonged to.

Numerous studies investigated the promotion of diversity to improve the overall workplace environment and quality of work (11). Gender diversity can improve the quality of healthcare, as it provides a flexible workplace environment for both men and women and healthcare services with diverse values (12). Moreover, gender differences in healthcare delivery allow for more patient-centered communication (13), guideline adherence (14), and testing for patients by female doctors (15).

### Importance of Role Models

More male than female cardiologists in this study had role models. Female cardiologists with a same-gender role model accounted only for 54% of the total, which may be due to a lower rate of female mentors (16), while male cardiologists accounted for 70%. Role models of the same gender are considered more appreciated for female cardiologists, as work situations for women are disproportionately influenced by marriage, childcare, and nursing. Otherwise, male role models also play an important role on female cardiologists because they can offer valuable guidance on many aspects of career development. To find role models of both genders, the construction of network systems, such as mentor–mentee matching on the national or worldwide scale is being awaited (17).

### Initiatives of the Committee for Diversity Promotion Committee of the Japanese Circulation Society

**FIGURE 6:**
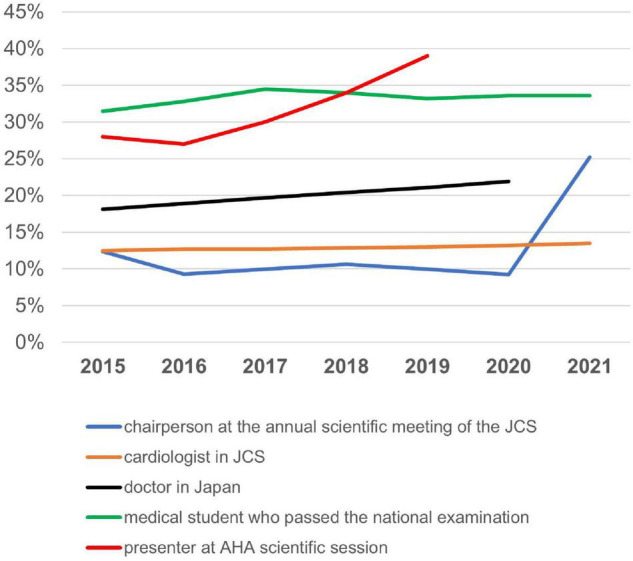
Changes in rates of female cardiologists. The changes in female cardiologist rates of female chairpersons at the annual scientific meeting of the JCS, female cardiologists in JCS, female doctors in Japan, female medical students who passed the national examination for medical practitioners, and female presenters at the AHA scientific session. JCS, Japan Circulation Society; AHA, American Heart Association.

To encourage female cardiologists, the JCS–JJC subcommittee was formed to increase proportion for chairing session in annual academic meetings (Figure 6). To meet the demands of a growing number of female doctors, future policies should improve the work environment of female cardiologists, provide support for research, increase the number of female doctors in instructive positions, allowing them to become role models, and strengthen communities with active communication using IT.

### Limitations

This study had some limitations. People who answered the questionnaire may have been more interested in chairing sessions than those who did not respond, which may differ from the usual population within an academic society.

## Conclusion

This study revealed that female cardiologists were less likely to accept chairing sessions compared with male cardiologists and that the presence of female cardiovascular specialists in the hospital positively influenced chair acceptance among both genders. Active and targeted promotion for this population would contribute to the revitalization of the field of cardiology through the empowerment of human resources.

## Data Availability Statement

The raw data supporting the conclusions of this article will be made available by the authors, without undue reservation.

## Ethics Statement

The studies involving human participants were reviewed and approved by this study design was approved by the JCS Ethics Committee (ID: 14). Written informed consent for participation was not required for this study in accordance with the national legislation and the institutional requirements.

## Author Contributions

AN, CK, SK, TI, YB, and YT planned and conducted the study. AN wrote the manuscript. YU analyzed the data. All authors contributed to data collection, analysis, interpretation, and approved the final version of the manuscript.

## Conflict of Interest

The authors declare that the research was conducted in the absence of any commercial or financial relationships that could be construed as a potential conflict of interest.

## Publisher’s Note

All claims expressed in this article are solely those of the authors and do not necessarily represent those of their affiliated organizations, or those of the publisher, the editors and the reviewers. Any product that may be evaluated in this article, or claim that may be made by its manufacturer, is not guaranteed or endorsed by the publisher.
